# CERA Attenuates Kidney Fibrogenesis in the *db*/*db* Mouse by Influencing the Renal Myofibroblast Generation

**DOI:** 10.3390/jcm7020015

**Published:** 2018-01-30

**Authors:** Christin Fischer, Natalie Deininger, Gunter Wolf, Ivonne Loeffler

**Affiliations:** 1 Department of Internal Medicine III, University Hospital Jena, Am Klinikum 1, D-07747 Jena, Germany; fischer.christin@uni-jena.de (C.F.); Gunter.Wolf@med.uni-jena.de (G.W.); 2 Institute of Medical Genetics and Applied Genomics, University Tübingen, Calwerstraße 7, D-72076 Tübingen, Germany; Natalie.Deininger@web.de

**Keywords:** diabetic nephropathy, DN, erythropoietin, EPO, CERA, tubulointerstitial fibrosis, TIF, myofibroblast, type 2 diabetes, *db*/*db* mouse

## Abstract

Tubulointerstitial fibrosis (TIF) is a pivotal pathophysiological process in patients with diabetic nephropathy (DN). Multiple profibrotic factors and cell types, including transforming growth factor beta 1 (TGF-β1) and interstitial myofibroblasts, respectively, are responsible for the accumulation of extracellular matrix in the kidney. Matrix-producing myofibroblasts can originate from different sources and different mechanisms are involved in the activation process of the myofibroblasts in the fibrotic kidney. In this study, 16-week-old *db*/*db* mice, a model for type 2 DN, were treated for two weeks with continuous erythropoietin receptor activator (CERA), a synthetic erythropoietin variant with possible non-hematopoietic, tissue-protective effects. Non-diabetic and diabetic mice treated with placebo were used as controls. The effects of CERA on tubulointerstitial fibrosis (TIF) as well as on the generation of the matrix-producing myofibroblasts were evaluated by morphological, immunohistochemical, and molecular biological methods. The placebo-treated diabetic mice showed significant signs of beginning renal TIF (shown by picrosirius red staining; increased connective tissue growth factor (CTGF), fibronectin and collagen I deposition; upregulated KIM1 expression) together with an increased number of interstitial myofibroblasts (shown by different mesenchymal markers), while kidneys from diabetic mice treated with CERA revealed less TIF and fewer myofibroblasts. The mechanisms, in which CERA acts as an anti-fibrotic agent/drug, seem to be multifaceted: first, CERA inhibits the generation of matrix-producing myofibroblasts and second, CERA increases the ability for tissue repair. Many of these CERA effects can be explained by the finding that CERA inhibits the renal expression of the cytokine TGF-β1.

## 1. Introduction

Diabetes mellitus (DM) is characterized by chronic hyperglycemia and within the two primary forms of DM, type 1 and type 2 (T1DM and T2DM), the non-insulin-dependent T2DM is the most common form of diabetes and is steadily increasing in developed countries [1]. T2DM patients are more susceptible to different complications, such as cardiovascular disease, diabetic neuropathy, retinopathy, and nephropathy [1]. Diabetic nephropathy (DN) is one of the most important microvascular complications, whose earliest manifestation is the presence of minute amounts of urinary protein (microalbuminuria) [1]. DN occurs in approximately 20–40% of patients with diabetes and is the leading cause of chronic kidney diseases and of end-stage renal disease in Europe and the U.S. [2,3]. Although albuminuria is classically regarded as the consequence of diabetes-induced glomerular damage, it is increasingly appreciated that the renal tubulointerstitium also plays an important role in the pathogenesis of DN [3]. All patients with chronic kidney disease show a progressive decline in renal function with time and the tubulointerstitial fibrosis (TIF) is a key cause of this pathophysiology [4]. Fibrogenesis, so-called scarring, involves an excess accumulation of extracellular matrix (ECM), which is a complex mixture of fibronectin, elastin, laminin, and collagens [4]. Renal mesenchymal cells are the principal matrix-producing cells and are responsible for the accumulation and remodeling of ECM in the kidney [4,5]. At least six distinct cell types with mesenchymal phenotypes can be detected in the kidney: (i) activated myofibroblasts; (ii) resident interstitial fibroblasts; (iii) bone marrow-derived fibrocytes; (iv) differentiated vascular pericytes; (v) tubular cells after epithelial-to-mesenchymal transition (EMT); and (vi) endothelial cells after endothelial-to-mesenchymal transition (EndMT) [6]. The latter two are mechanisms, in that epithelial or endothelial cells undergo biochemical changes, which lead to a matrix-producing mesenchymal phenotype. EMT and EndMT are highly regulated processes and are defined by four key steps: (i) loss of epithelial or endothelial characteristics; (ii) de novo α-SMA (alpha-smooth muscle actin) expression and actin reorganization; (iii) disruption of basement membranes under the control of matrix metalloproteinases; and (iv) enhanced cell migration and invasion of the interstitium [7,8,9,10,11,12,13,14]. Several molecular factors are well known to mediate fibrogenesis in DN in a stage-dependent manner [12]. Uncontrolled high glucose concentrations in diabetes induce the production of various profibrotic molecules with transforming growth factor β1 (TGF-β1) as the major player in the induction and progression of TIF [4,12]. The *db*/*db* mice, an experimental model for T2DM, have a mutation deletion of the leptin receptor, and leptin deficiency confers susceptibility to obesity and insulin resistance [15]. The underlying genetic background is susceptible to diabetic complication such as nephropathy, and DN in these mice is manifested by albuminuria, due to podocyte loss, and mesangial matrix expansion but also signs of TIF [15,16]. A number of potential therapeutic strategies are being employed to ameliorate progression of TIF. In addition to indirect key strategies (e.g., reduction of hyperglycemia), increasing evidence suggests that erythropoietin (EPO), independent from its hematopoietic effect, may be protective for several tissues, including the heart, brain, and kidney, which may be useful in the prevention of renal disease [17]. The kidney-protective potential of different EPO molecules (e.g., epoetin-β, darbepoetin-β, and continuous erythropoietin receptor activator (CERA)) has been shown in several studies [18,19,20,21]. CERA, a long acting erythropoiesis stimulating agent in treating renal anaemia in patients with diabetic chronic kidney disease, has been shown to ameliorate increased albuminuria by preventing diabetes-induced podocyte damage in the *db*/*db* mouse which was apart from hematopoietic effects and without influencing the hyperglycemia, but rather by EPO receptor activation on renal cells [18,20,21].

However, little is known about the possible protective effect of CERA on beginning TIF in *db*/*db* mice. In the light of this background, in this study we evaluated different markers for TIF and sources of myofibroblasts in diabetic *db*/*db* mice after treatment with placebo or CERA.

## 2. Materials and Methods

### 2.1. Animals

All animal experiments were approved by the local ethics committee, and were performed in accordance with the German Animal Protection Law and are described previously [18]. Briefly, the *db*/*db* (B6.Cg-*Dock7^m^ Lepr^db^*/++/J) animals (*n* = 8) were treated with 1.2 μg/kg i.p. CERA (continuous erythropoietin receptor activator, MIRCERA, Hoffmann-La Roche, Grenzach-Wyhlen, Germany) once per week for 2 weeks. Non-diabetic (*n* = 8) and diabetic *db*/*db* mice (*n* = 10) were injected with 0.9% i.p. NaCl (placebo) as control. All mice were 14–16 weeks old at the beginning of the study and only male mice were used to control for potential hormonal effects.

### 2.2. Picrosirius Red Staining

For evaluation of the kidney fibrosis paraffin-embedded, 2 μm sections were stained with Sirius Red after Puchtler et al. [22,23]. The paraffin sections were de-waxed, hydrated, and stained with picrosirius red solution (0.1% Sirius Red F3B in saturated picric acid) for 1 h, washing twice in acidified water, dehydrating, and then clearing in xylene. The tissue sections were counterstained with Weigert’s iron hematoxylin solution for 5 min (Morphisto Lab, Frankfurt, Germany). Sirius red sections were viewed with bright field as well as with polarization contrast illumination at 20×. The collagen content was assessed according to [24], with assessment of the color changes of collagen fibers under polarized light: when fiber thickness increases, the color changes from green to yellow to orange to red.

### 2.3. Immunohistochemical Staining

Paraffin kidney sections (thickness 2 μm) from all animals were deparaffinized, exposed to heat-mediated antigen retrieval in citrate buffer (pH 6.0) (except for connective tissue growth factor (CTGF)) and then incubated with 3% H_2_O_2_ (Carl Roth GmbH & Co.KG, Karlsruhe, Germany) for 10 min at room temperature to block endogenous peroxidase. The following primary antibodies were used: rabbit anti-bone morphogenetic protein-7 (BMP-7), rabbit anti-cluster of differentiation 34 (CD34), rabbit anti-collagen I, rabbit anti-CTGF, rabbit anti-fibronectin, rabbit anti-fibroblast specific protein 1 (FSP1), rabbit anti-Ki67, rabbit anti-matrix metalloproteinase 2 (MMP2), rabbit anti-α-SMA, and goat anti-Snail1 (Abcam, Cambridge, UK), rabbit anti-vimentin (Epitomics, Burlingame, CA, USA), rabbit anti-E-cadherin, rabbit anti-TGF-β1 (Cell Signaling, Danvers, MA, USA). After incubation of the sections with peroxidase-labeled rabbit anti-goat or goat anti-rabbit IgG antibody (KPL, Gaithersburg, MD, USA), diaminobenzidine (DAB) was used as a chromogen (Peroxidase substrate Kit DAB (Vector Laboratories, Burlingame, CA, USA)). Except for the nuclear Ki67 and Snail1 staining, the tissue sections were counterstained with hemalum.

For imaging, documentation and quantification, five non-overlapping pictures (magnification 200×) per section were taken under standardized conditions using the AxioVision 4.8 software and the monochrome modus of the camera (AxioCam HRc; both Zeiss, Jena, Germany). Semi-quantitative analyses were performed by three independent investigators blinded to the origin of the groups using a semi-quantitative scoring system for the quantification of collagen I, CTGF, E-cadherin, fibronectin, and MMP2 expression, as well as of the tubular and endothelial expression of FSP1, α-SMA, and vimentin with: 0 = no staining, 1 = <10% of tubuli/vessels per field, 2 = 25% of tubuli/vessels per field, 3 = 50% of tubuli/vessels per field, 4 = 75% of tubuli/vessels per field, and 5 = 100% of tubuli/vessels per field [25,26]. The expression of Snail1, Ki67, CD34, and interstitial FSP1, α-SMA, and vimentin were quantified by counting the number of positive nuclei or interstitial cells per field. To combine the results from the three independent investigators, the values from the non-diabetic controls were assigned as an arbitrary value of 1.

### 2.4. RNA Isolation and Real-Time PCR

To determine the steady-state mRNA levels of collagen type I, KIM1 (kidney injury molecule 1) and TGF-β1, the total RNA was isolated from kidney homogenates using the RNeasy Lipid Tissue Mini Kit (Qiagen, Hilden, Germany) (*n* = 3 of the non-diabetic placebo group; *n* = 4 of the diabetic placebo group; *n* = 3 of the diabetic CERA group). DNA contaminations were eliminated using the RNase-Free DNase Set (Qiagen) and 1 μg total RNA was reverse-transcribed using the Reverse Transcription System from Promega (Madison, WI, USA). The expression levels of genes were determined as previously described [27]. Values were normalized to the average of GUSB (glucuronidase beta) and HPRT1 (hypoxanthine phospho-ribosyltransferase 1) expression, and the non-diabetic controls were assigned as an arbitrary value of 1. Primers were purchased from TIB Molbiol (Berlin, Germany) and the sequences and annealing temperature are listed in Table 1.

### 2.5. Analysis of Renal TGF-β1 Protein Expression

Total protein from the cortices of mouse kidneys (*n* = 3 per group) was isolated by homogenization with complete Lysis-M buffer supplemented with protease inhibitors (both from Roche Diagnostics, Mannheim, Germany). After centrifugation, the activated TGF-β1 concentrations in the protein lysates of the renal tissue were quantitatively determined by using the Mouse/Rat/Porcine/Canine TGF-β1 ELISA according to the manufacturer’s instructions (Quantikine, R&D Systems, Wiesbaden, Germany). The concentration of TGF-β1 in the kidney was expressed as picograms per milligram of total protein.

### 2.6. Statistical Analyses

All data in this article are reported as means ± SEM. Statistical analysis was performed using SPSS statistics (IBM, Armonk, NY, USA). Results were analyzed using the Kruskal-Wallis test for multi-group comparisons, followed by the Mann-Whitney rank sum test to compare individual groups of mice. Statistical significance was accepted at *p* ≤ 0.05.

## 3. Results and Discussion

### 3.1. CERA Inhibits Tubulointerstitial Fibrosis in Mice with Diabetes Type 2

**Figure 1:**
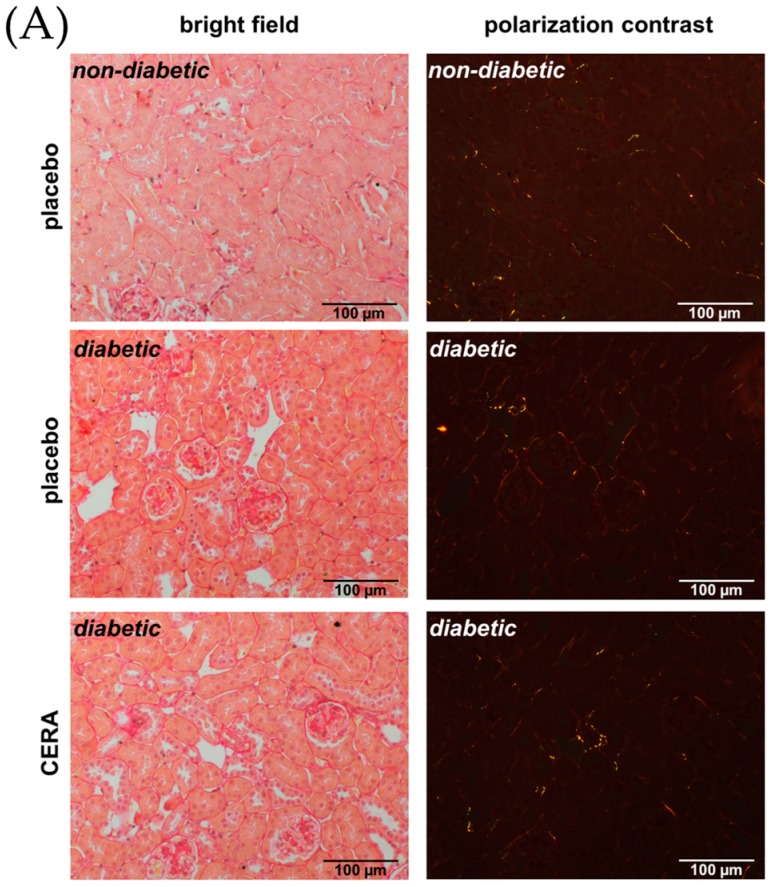

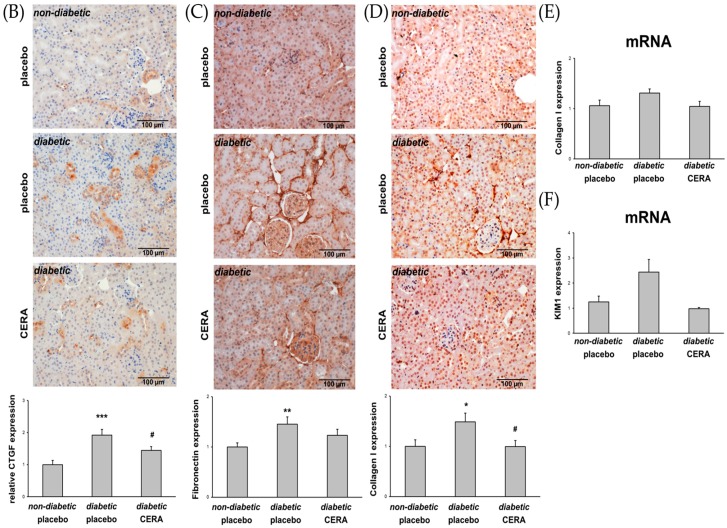
The tubulointerstitial fibrosis is reduced by CERA in diabetic kidneys. (**A**) Picrosirius red. Representative images of picrosirius red staining (magnification: 200×). Left: bright field illumination. Right: polarization contrast illumination. The color of the fibers shows the collagen content (increasing thickness: from green to yellow to orange to red); (**B**) CTGF. Representative images of CTGF immunohistochemistry (magnification: 200×) and semi-quantitative analysis of the staining (graph); (**C**) Fibronectin. Representative images of fibronectin immunohistochemistry (magnification: 200×) and semi-quantitative analysis of the staining (graph); (**D**) Collagen I. Representative images of collagen type I immunohistochemistry (magnification: 200×) and semi-quantitative analysis of the staining (graph); (**E**) Collagen I mRNA. Real-time PCR analysis of the renal collagen I mRNA expression. Values were normalized to the expression of housekeeping genes; (**F**) KIM1 mRNA. Real-time PCR analysis of the renal KIM1 mRNA expression. Values were normalized to the expression of housekeeping genes. Whereas placebo-treated diabetic animals show significant increase in all tested fibrosis marker, compared to non-diabetic controls, CERA treatment reveals anti-fibrotic effects in kidneys of diabetic mice. * *p* ≤ 0.05, ** *p* ≤ 0.01, *** *p* ≤ 0.001 = diabetic vs. non-diabetic control; ^#^ *p* ≤ 0.05 = *db*/*db* CERA vs. *db*/*db* placebo. The non-diabetic controls were assigned as an arbitrary value of 1.

Recently, we demonstrated that medication of overt DN with CERA for a short time could ameliorate albuminuria and podocyte loss in *db*/*db* mice [18]. Of particular importance, the shown kidney protective action of CERA was independent from the well-known hematopoietic effect of EPO [18]. This suggests that EPO or its analogs can mediate their positive impact also via direct signaling through the EPO receptor on the surface of the renal cells [18]. Among glomerular pathology, the rate of deterioration of renal function correlates best with the degree of TIF in DN [33]. Therefore, we studied the effect of CERA on TIF in genetic-induced diabetic kidney injury in *db*/*db* mice (Figure 1). Several different morphometry techniques have been employed to assess fibrosis, including Masson trichrome, Sirius red and immunohistochemistry for fibronectin and collagens [34]. Visual assessment using Masson trichrome stained slides is the standard practice, but has been reported to be poorly reproducible, whereas repeated analysis is highly consistent for Sirius red staining, which is specific for collagen types I and III fibrils under polarized light [34,35]. Figure 1A shows the picrosirius red staining with unpolarized light (left images) and the appropriate polarization contrast microscopy (right images). It proved difficult to quantify differences in the collagen content or fiber thickness between non-diabetic and diabetic animals and much less between placebo- and CERA-treated diabetic mice from brightfield images. Therefore, we calculated the collagen content by analysis of collagen fiber color following Rich et al. [24]. The color of picrosirius red-stained collagen seen with polarized light changes as fiber thickness increases: from green (thin) to yellow and orange up to red (thick) [24]. Whereas kidney sections of non-diabetic mice show almost no or at most yellow-stained fibers, an appearance of orange- or red-stained fibers can be observed in diabetic kidneys (Figure 1A right panel). This indicates an increase in collagen content and fiber thickness as a measure for beginning TIF. CERA attenuates the diabetes-induced thickening of collagen fibers as the proportion of interstitial red fibers decreased, while the proportion of orange and yellow fibers increased compared with kidneys from placebo-treated diabetic mice (Figure 1A right panel). To support this finding, we investigated the synthesis and accumulation of the fibrosis inductor and marker connective tissue growth factor (CTGF) as well as the matrix molecules fibronectin and collagen type I (Figure 1B–E). CTGF is a profibrotic cytokine and has been shown to be involved in both the early and later stages of DN, where it is strongly induced [36]. Downstream of a cascade of events induced by hyperglycemia, CTGF promotes increased expression of ECM proteins (e.g., collagen type I, and fibronectin) [36]. Although, CTGF is a downstream mediator of the profibrotic signaling of TGF-β1, it has also TGF-β-independent effects to enhance renal fibrosis: it has been shown that renal tubular expression of CTGF correlates with TIF in DN and that specific down-regulation of CTGF attenuates nephropathy in mouse models of type 1 and 2 DN [37]. The adhesive glycoprotein fibronectin is the major ECM protein and during fibrogenesis initially produced, serves as a scaffold for the deposition of other proteins, such as collagen type I and III, and is thought to function as a fibroblast chemoattractant to amplify the fibrotic response [7,38]. As expected, the semi-quantitative assessment of these fibrosis-related proteins demonstrated a significant enhanced expression under diabetic conditions (Figure 1B–D). In contrast, when diabetic mice are treated with CERA, the production or deposition of CTGF and the matrix molecules fibronectin and collagen I is diminished (Figure 1B–D). Our data further show that CERA decreases the diabetes-induced renal expression of kidney injury molecule 1 (KIM1), a transmembrane tubular protein with unknown function, to the level of non-diabetic controls (Figure 1F). It has been published that KIM1 is significantly increased in DN and is used as a new biomarker for tubular injury and indirectly for TIF, because it is primarily expressed at the luminal side of dedifferentiated proximal tubules, in areas with fibrosis [25,39].

Taken together, our data suggest that diabetic mice with an age of 16 weeks begin to develop early TIF, whereas CERA seems to inhibit the glucose-induced interstitial fibrogenesis. This is, in addition to our previously demonstrated protective effects of CERA on glomerular pathology [18], a further explanation for the ameliorated decrease in renal function by CERA.

### 3.2. CERA Influences Myofibroblast Population in Renal Interstitium of db/db Mice

**Figure 2:**
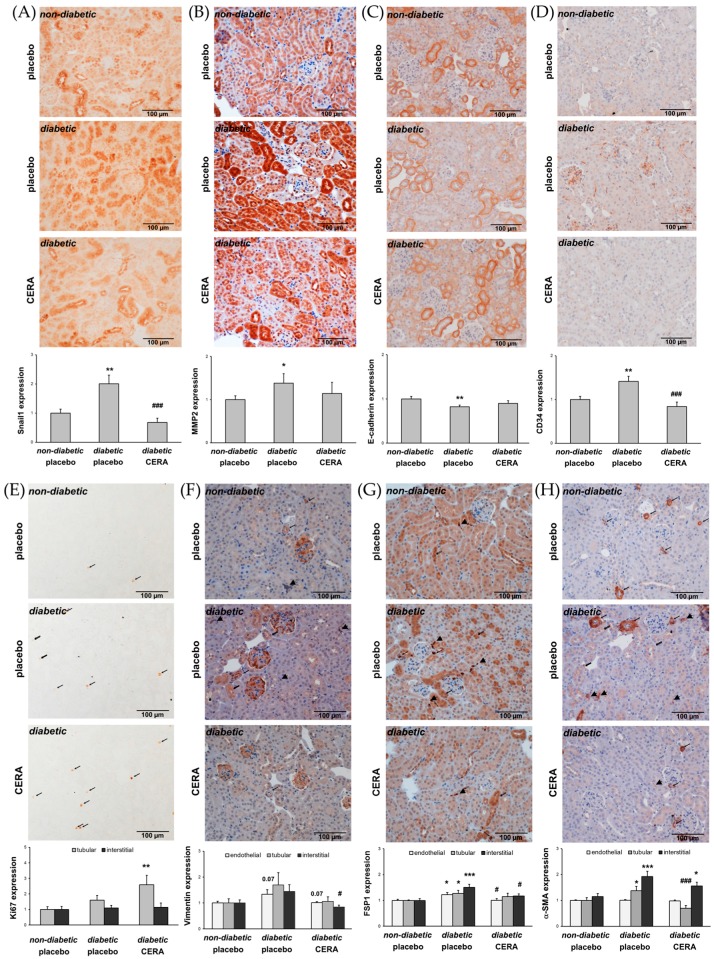
The sources of interstitial myofibroblasts are influenced in *db*/*db* mice. (**A**) Snail1. Representative images of Snail1 immunohistochemistry (magnification: 200×) and semi-quantitative analysis of the number of positively stained cells (graph); (**B**) MMP2. Representative images of MMP2 immunohistochemistry (magnification: 200×) and semi-quantitative analysis of the staining (graph); (**C**) E-cadherin. Representative images of E-cadherin immunohistochemistry (magnification: 200×) and semi-quantitative analysis of the staining (graph); (**D**) CD34. Representative images of CD34 immunohistochemistry (magnification: 200×) and semi-quantitative analysis of the number of positively stained interstitial cells (graph); (**E**) Ki67. Representative images of Ki67 immunohistochemistry (magnification: 200×) and semi-quantitative analysis of the number of positively stained nuclei of tubular (thin arrow) and interstitial cells (double thin arrow) (graph); (**F**) Vimentin. Representative images of vimentin immunohistochemistry (magnification: 200×) and semi-quantitative analysis of the endothelial and tubular staining as well as the number of positively stained interstitial cells (graph). Positive endothelial cells (thin arrow), positive tubular cells (double thin arrow), and positive interstitial cells (thick arrow); (**G**) FSP1. Representative images of FSP1 immunohistochemistry (magnification: 200×) and semi-quantitative analysis of the endothelial and tubular staining as well as the number of positively stained interstitial cells (graph). Positive endothelial cells (thin arrow), positive tubular cells (double thin arrow), and positive interstitial cells (thick arrow); (**H**) α-SMA. Representative images of α-SMA immunohistochemistry (magnification: 200×) and semi-quantitative analysis of the endothelial and tubular staining as well as the number of positively stained interstitial cells (graph). Positive endothelial cells (thin arrow), positive tubular cells (double thin arrow), and positive interstitial cells (thick arrow). The interstitial matrix-producing myofibroblasts are reduced in diabetic kidneys when treated with CERA. This is partly due to reduction of EMT-like or EndMT-like changes and less migration of fibrocytes into the interstitium. CERA also increases the proliferation rate of tubular cells. * *p* ≤ 0.05, ** *p* ≤ 0.01, *** *p* ≤ 0.001 = diabetic vs. non-diabetic control; ^#^ *p* ≤ 0.05, ^###^ *p* ≤ 0.001 = *db*/*db* CERA vs. *db*/*db* placebo. The non-diabetic controls were assigned as an arbitrary value of 1.

The percentage of myofibroblasts is proportionally correlated with the severity of renal fibrosis [40]. Myofibroblasts may arise from a number of sources such as activated renal fibroblasts, EMT, EndMT, and bone marrow-derived fibrocytes [4,40,41]. To delineate the origin of the renal myofibroblasts in diabetic *db*/*db* mice, we stained the kidney sections with several markers and assessed the changes in diabetic kidneys compared to non-diabetic controls as well as the influence of CERA on the observed changes (Figure 2).

Snail1, a transcription factor that can be activated by TGF-β pathways but also in a TGF-β-independent manner, controls major biological processes responsible for renal fibrogenesis, including mesenchymal reprogramming of tubular epithelial or endothelial cells [13,14,42]. Our results from the assessment of Snail1 expression (Figure 2A) are consistent with the findings that in renal biopsies of patients with DN as well as in kidneys of type 2 diabetic mice, the level of Snail1 are upregulated, which is associated with enhanced EMT/EndMT-like changes and TIF [14,16,43]. Snail1, as the most prominent EMT transcriptional regulator, is known to induce MMP2 and downregulate E-cadherin initiating EMT [44,45,46,47]. Here we demonstrate, that placebo-treated diabetic mice exhibit enhanced MMP2 expression (Figure 2B) as well as significantly decreased E-cadherin (Figure 2C) compared with age-matched non-diabetic animals, suggesting that EMT-like changes possibly play a role in renal fibrogenesis at this time point of disease. Although, for the purpose of EMT, Snail upregulates MMP2 to degrade the basement membrane [48], it has also been shown that enhanced expression of MMP2 represents a primary mediator of renal injury and reduced MMP2 expression protects mice against renal fibrosis, as opposed to the generally accepted concept that limits the pathogenic role of MMP2 activity to the phase of epithelial cell migration/invasion into the interstitial space [49,50]. In addition to the EMT and EndMT processes, in chronic injury, bone marrow-derived fibrocytes can also be a source of matrix-producing myofibroblasts [4,41]. Our data supports this hypothesis, as the number of interstitial CD34^+^ cells is significantly increased in diabetic mice with TIF (Figure 2D). In answer to the question of the CERA effects on the observed TIF-promoting events, it is revealed that CERA reversed these pathological processes: the increase of Snail1 and MMP2, the decrease of E-cadherin, as well as the invasion of bone marrow-derived fibrocytes (Figure 2A–D). In addition to its role as a local regulator of EMT/EndMT, interestingly, paracrine, “secreted” Snail1 may also contribute to the recruitment or proliferation of interstitial myofibroblasts after kidney injury in a more global process [51,52,53,54]. To determine the proliferation of tubulointerstitial cells, as a further source of myofibroblasts, we performed immunohistochemical staining of kidney sections from non-diabetic as well as diabetic mice with the cell division marker Ki67 (Figure 2E). Only sporadic proliferating Ki67-positive cells were observed in the normal kidneys of healthy animals, which was also shown by other groups [55]. Contrary to our expectations, the semi-quantitative assessment of the Ki67 positive-stained cells showed no substantial increase of cell proliferation in the interstitium in diabetic kidneys independent from treatment (Figure 2E), suggesting that, at least at this stage of renal fibrogenesis, the proliferation of resident fibroblasts plays no or a minor role for the generation of activated myofibroblasts. Interestingly, the result of cell counting of proliferating tubular epithelial cells is a slight increase under diabetic conditions, which is significantly intensified after administration of CERA (Figure 2E). It has been previously described, that epithelial cells, when damaged in the proximal portion of the nephron, rapidly proliferate to repair the damage to the kidney [4,39]. The kidney has a good capacity for regeneration after injury and fibrosis is thought to result from wound healing processes that fail to terminate [4,56]. Unfortunately, while much is known about the contribution of various molecules and signaling pathways to this “repair” process, little is known about what eventually goes wrong [56]. 

As described above, EMT and EndMT are defined by different key steps, including the gain of mesenchymal cell marker, such as vimentin and fibroblast specific protein 1 (FSP1), and the α-SMA de novo synthesis, whose expression we investigated in diabetic mice treated with placebo or CERA and non-diabetic mice (Figure 2F–H). Although, we found a numerical or significant upregulation of vimentin (Figure 2F) and FSP1 (Figure 2G) in endothelial as well as tubular cells under diabetic conditions, a substantial and evaluable de novo synthesis of α-SMA (Figure 2H), was only found in tubular cells, which indicates that rather EMT-like changes than full-way EndMT occurs in this stage of diabetes-induced fibrogenesis. However, equally to the initial EMT events, shown in Figure 2A–C, these consequent key steps of EMT are also reversed by CERA treatment (Figure 2F–H). In addition to the evaluation of the endothelial and tubular expression of vimentin, FSP1 and α-SMA, we counted the number of interstitial cells, which are positive for these markers (Figure 2F–H). We found a distinct upregulation of tubulointerstititial cells and an attenuation by CERA, no matter what we have analyzed. Due to the diverse origin and mixed phenotypic heterogeneity of myofibroblasts as well as to the relative lack of specific markers [6,40], it is challenging to distinguish the one cell type from the other. For example, interstitial myofibroblasts under pathological conditions are positive for all of the three markers (α-SMA, vimentin and FSP1), while vimentin also stains interstitial pericytes (in addition positive for α-SMA, but negative for FSP1) and resident fibroblasts (in addition positive for FSP1, but negative for α-SMA) [39,57].

Taken together, although, owing to the overlap of marker expression, only ultrastructural analysis can discriminate between the different interstitial cell types [57], our data indicate that CERA has a potent protective property against myofibroblast generation on the one hand, and increases the renal capacity for regeneration, on the other hand.

### 3.3. The Expression of TGF-β1 Is Reduced by CERA in Diabetic Kidneys

**Figure 3:**
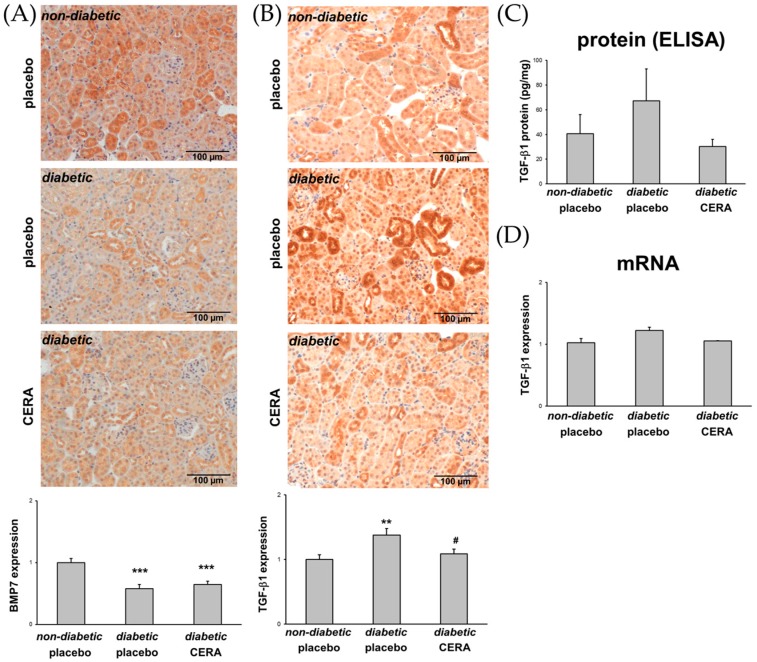
Analyses of CERA effects on the renal expression of the TGF-β superfamily members BMP7 and TGF-β1. (**A**) BMP7. Representative images of BMP7 immunohistochemistry (magnification: 200×) and semi-quantitative analysis of the staining (graph); (**B**) TGF-β1. Representative images of TGF-β1 immunohistochemistry (magnification: 200×) and semi-quantitative analysis of the staining (graph); (**C**) TGF-β1 protein. Activated TGF-β1 protein levels in lysates of the renal tissue. The TGF-β1 concentration in the kidney is expressed as pg/mg of total protein; (**D**) TGF-β1 mRNA. Real-time PCR analysis of the renal TGF-1 mRNA expression. Values were normalized to the expression of housekeeping genes. The inhibition of the BMP7 expression in diabetic kidneys, compared to healthy controls, is not influenced by CERA treatment. Whereas diabetic kidneys express increased amounts of pro-fibrotic TGF-β1, the diabetic animals that are treated with CERA show less TGF-β1 mRNA as well as protein in their renal tissue. ** *p* ≤ 0.01, *** *p* ≤ 0.001 = diabetic vs. non-diabetic control; ^#^ *p* ≤ 0.05 = *db*/*db* CERA vs. *db*/*db* placebo. The non-diabetic controls were assigned as an arbitrary value of 1 (except in (**C**)).

In type 1 and 2 diabetes, the broad-spectrum cytokine TGF-β1 is increased and regulates many biological processes [58,59,60,61]. The TGF-β1-mediated effects influence the pathology of different renal cells, which subsequently leads to TIF [58,62]. BMP7, a homodimeric member of the TGF-β superfamily, is primarily expressed in kidney tubules and glomeruli [63]. Physiologically, BMP7 plays an important role in the kidney development and regulation of nephrogenesis, but in human and experimental DN, the renal cortical expression of BMP7 is progressively decreased [64,65]. Several studies demonstrated, that BMP7 can counteract the TGF-β1 action by inhibiting TGF-β-mediated fibrosis and TGF-β-induced EMT/EndMT [14,45,64,66,67,68]. To investigate whether the renoprotective effect of CERA is due to the influence of the BMP7 expression, we first evaluated the renal expression of BMP7 (Figure 3A). Indeed, we could confirm that the levels of renal BMP7 are significantly reduced under diabetic conditions, but administration of CERA could not reverse the downregulation of BMP7. Finally, we analyzed the renal TGF-β1 protein and mRNA and, in fact, in kidneys from diabetic mice treated with CERA the upregulation of TGF-β1 was no longer detectable (Figure 3B–D), indicating that CERA exhibits the potential to inhibit the profibrotic TGF-β1.

This finding presents a good explanation for at least the CERA-provoked attenuation of TIF and EMT-like changes. Very recently, we have also suggested that the significantly reduced clinical signs of DN (less renal TIF as well as EMT-like changes) are most likely due to the decreased TGF-β1 expression by upregulated EPO in kidneys of diabetic transgenic mice in which the HIF/EPO axis is activated [16]. The question is: how can EPO/CERA exert this capacity to influence the TGF-β1 expression? CERA may inhibit the TGF-β1-mediated signaling as well as the TGF-β1 expression itself, which is based on studies that have demonstrated, that TGF-β1 induces its own expression in a pSmad3-dependent manner in renal cells [69] and that EPO treatment suppresses TGF-β1-mediated signaling by inhibiting the phosphorylation and nuclear translocation of Smad3 [70]. In addition, Zhang et al. have shown that TGF-β1 activates NF-κB via ERK MAP kinase and that this is required for transcriptional autoinduction of TGF-β1 [71]. While erythropoiesis is stimulated by the canonical EpoR homodimer, the tissue-protective effects of EPO are mediated through a heterodimeric “tissue-protective” receptor. This tissue-protective receptor is supposed to be a heterocomplex composed of EpoR and the ubiquitous β-common receptor (βcR, CD131, colony-stimulating factor 2 receptor-β) [72]. Both, the activation of βcR and the activation of Akt are essential for renoprotection by EPO [73]. It has been demonstrated that EPO enhances, in a βcR-dependent manner, the activation of Akt, resulting in inhibition of activation of NF-κB [73].

## 4. Conclusions

This study demonstrates the non-hematopoietic, tissue-protective effect of CERA on beginning TIF in *db*/*db* mice with type 2 DN. This is due to both the inhibition of the generation of matrix-producing interstitial myofibroblasts and the enhanced ability for tissue repair after treatment of diabetic mice with CERA. Most of these kidney-protective effects are the results of the CERA-mediated attenuation of the renal expression of TGF-β1, the maybe most potent fibrotic cytokine. Further investigations are needed to understand the mechanisms by which CERA may influence kidney repair and regeneration.

## Figures and Tables

**Table 1:** Primer sequences and annealing temperature used for real-time PCR.

Target	Sence Primer	Antisense Primer	T_Ann._	Reference
collagen I	5′-GAGAGGTGAACAAGGTCCCG-3′	5′-AAACCTCTCTCGCCTCTTGC-3′	64 °C	[28]
KIM1	5′-ATGAATCAGATTCAAGTCTTC-3′	5′-TCTGGTTTGTGAGTCCATGTG-3′	55 °C	[29]
TGF-β1	5′-AAGGGCTACCATGCCAACTT-3′	5′-CGGGTTGTGTTGGTTGTAGA-3′	62 °C	[30]
GUSB	5′-CCGATTATCCAGAGCGAGTATG-3′	5′-CTCAGCGGTGACTGGTTCG-3′	59 °C	[31]
HPRT	5′-TGGATACAGGCCAGACTTTGTT-3′	5′-CAGATTCAACTTGCGCTCATC-3′	59 °C	[32]

## References

[1] Wu Y., Ding Y., Tanaka Y., Zhang W. (2014). Risk factors contributing to type 2 diabetes and recent advances in the treatment and prevention. Int. J. Med. Sci..

[2] Molitch M.E., DeFronzo R.A., Franz M.J., Keane W.F., Mogensen C.E., Parving H.H., Steffes M.W., American Diabetes Association (2004). Nephropathy in diabetes. Diabetes Care.

[3] Nauta F.L., Boertien W.E., Bakker S.J., van Goor H., van Oeveren W., de Jong P.E., Bilo H., Gansevoort R.T. (2011). Glomerular and tubular damage markers are elevated in patients with diabetes. Diabetes Care.

[4] Hewitson T.D. (2009). Renal tubulointerstitial fibrosis: Common but never simple. Am. J. Physiol. Renal Physiol..

[5] Simonson M.S. (2007). Phenotypic transitions and fibrosis in diabetic nephropathy. Kidney Int..

[6] Strutz F., Zeisberg M. (2006). Renal fibroblasts and myofibroblasts in chronic kidney disease. J. Am. Soc. Nephrol..

[7] Qi W., Chen X., Poronnik P., Pollock C.A. (2006). The renal cortical fibroblast in renal tubulointerstitial fibrosis. Int. J. Biochem. Cell Biol..

[8] Yang J., Liu Y. (2001). Dissection of key events in tubular epithelial to myofibroblast transition and its implications in renal interstitial fibrosis. Am. J. Pathol..

[9] Liu Y. (2004). Epithelial to mesenchymal transition in renal fibrogenesis: Pathologic significance, molecular mechanism, and therapeutic intervention. J. Am. Soc. Nephrol..

[10] Kanasaki K., Taduri G., Koya D. (2013). Diabetic nephropathy: The role of inflammation in fibroblast activation and kidney fibrosis. Front. Endocrinol. (Lausanne).

[11] Zeisberg E.M., Potenta S.E., Sugimoto H., Zeisberg M., Kalluri R. (2008). Fibroblasts in kidney fibrosis emerge via endothelial-to-mesenchymal transition. J. Am. Soc. Nephrol..

[12] Loeffler I., Wolf G. (2015). Epithelial-to-mesenchymal transition in diabetic nephropathy: Fact or fiction?. Cells.

[13] Li J., Qu X., Bertram J.F. (2009). Endothelial-myofibroblast transition contributes to the early development of diabetic renal interstitial fibrosis in streptozotocin-induced diabetic mice. Am. J. Pathol..

[14] Kizu A., Medici D., Kalluri R. (2009). Endothelial-mesenchymal transition as a novel mechanism for generating myofibroblasts during diabetic nephropathy. Am. J. Pathol..

[15] Alpers C.E., Hudkins K.L. (2011). Mouse models of diabetic nephropathy. Curr. Opin. Nephrol. Hypertens..

[16] Loeffler I., Liebisch M., Daniel C., Amann K., Wolf G. (2017). Heterozygosity of mitogen-activated protein kinase organizer 1 ameliorates diabetic nephropathy and suppresses epithelial-to-mesenchymal transition-like changes in *db*/*db* mice. Nephrol. Dial. Transplant..

[17] Loeffler I., Wolf G. (2015). The role of hypoxia and Morg1 in renal injury. Eur. J. Clin. Investig..

[18] Loeffler I., Ruster C., Franke S., Liebisch M., Wolf G. (2013). Erythropoietin ameliorates podocyte injury in advanced diabetic nephropathy in the *db*/*db* mouse. Am. J. Physiol. Renal Physiol..

[19] Eren Z., Gunal M.Y., Ari E., Coban J., Cakalagaoglu F., Caglayan B., Beker M.C., Akdeniz T., Yanikkaya G., Kilic E. (2016). Pleiotropic and renoprotective effects of erythropoietin beta on experimental diabetic nephropathy model. Nephron.

[20] Menne J., Park J.K., Shushakova N., Mengel M., Meier M., Fliser D. (2007). The continuous erythropoietin receptor activator affects different pathways of diabetic renal injury. J. Am. Soc. Nephrol..

[21] Schiffer M., Park J.K., Tossidou I., Bartels J., Shushakova N., Menne J., Fliser D. (2008). Erythropoietin prevents diabetes-induced podocyte damage. Kidney Blood Press. Res..

[22] Puchtler H., Waldrop F.S., Valentine L.S. (1973). Polarization microscopic studies of connective tissue stained with picro-sirius red FBA. Beiträge zur Pathologie.

[23] Junqueira L.C., Bignolas G., Brentani R.R. (1979). Picrosirius staining plus polarization microscopy, a specific method for collagen detection in tissue sections. Histochem. J..

[24] Rich L., Whittaker P. (2005). Collagen and picrosirius red staining: A polarized light assessment of fibrillary hue and spatial distribution. Braz. J. Morphol. Sci..

[25] Van Timmeren M.M., van den Heuvel M.C., Bailly V., Bakker S.J., van Goor H., Stegeman C.A. (2007). Tubular kidney injury molecule-1 (KIM-1) in human renal disease. J. Pathol..

[26] Xu-Dubois Y.C., Galichon P., Brocheriou I., Baugey E., Morichon R., Jouanneau C., Ouali N., Rondeau E., Hertig A. (2014). Expression of the transcriptional regulator snail1 in kidney transplants displaying epithelial-to-mesenchymal transition features. Nephrol. Dial. Transplant..

[27] Hammerschmidt E., Loeffler I., Wolf G. (2009). Morg1 heterozygous mice are protected from acute renal ischemia-reperfusion injury. Am. J. Physiol. Renal Physiol..

[28] Lei W., Long Y., Li S., Liu Z., Zhu F., Hou F.F., Nie J. (2015). Homocysteine induces collagen I expression by downregulating histone methyltransferase G9a. PLoS ONE.

[29] Humphreys B.D., Xu F., Sabbisetti V., Grgic I., Movahedi Naini S., Wang N., Chen G., Xiao S., Patel D., Henderson J.M. (2013). Chronic epithelial kidney injury molecule-1 expression causes murine kidney fibrosis. J. Clin. Investig..

[30] Sahlberg C., Peltonen E., Lukinmaa P.L., Alaluusua S. (2007). Dioxin alters gene expression in mouse embryonic tooth explants. J. Dent. Res..

[31] Rauthan M., Pilon M. (2015). A chemical screen to identify inducers of the mitochondrial unfolded protein response in *C. elegans*. Worm.

[32] Costamagna D., Quattrocelli M., van Tienen F., Umans L., de Coo I.F., Zwijsen A., Huylebroeck D., Sampaolesi M. (2016). Smad1/5/8 are myogenic regulators of murine and human mesoangioblasts. J. Mol. Cell Biol..

[33] Phillips A.O., Steadman R. (2002). Diabetic nephropathy: The central role of renal proximal tubular cells in tubulointerstitial injury. Histol. Histopathol..

[34] Farris A.B., Adams C.D., Brousaides N., Della Pelle P.A., Collins A.B., Moradi E., Smith R.N., Grimm P.C., Colvin R.B. (2011). Morphometric and visual evaluation of fibrosis in renal biopsies. J. Am. Soc. Nephrol..

[35] Street J.M., Souza A.C., Alvarez-Prats A., Horino T., Hu X., Yuen P.S., Star R.A. (2014). Automated quantification of renal fibrosis with Sirius Red and polarization contrast microscopy. Physiol. Rep..

[36] Mason R.M., Wahab N.A. (2003). Extracellular matrix metabolism in diabetic nephropathy. J. Am. Soc. Nephrol..

[37] Bonventre J.V. (2012). Can we target tubular damage to prevent renal function decline in diabetes?. Semin. Nephrol..

[38] Qi W., Twigg S., Chen X., Polhill T.S., Poronnik P., Gilbert R.E., Pollock C.A. (2005). Integrated actions of transforming growth factor-beta1 and connective tissue growth factor in renal fibrosis. Am. J. Physiol. Renal Physiol..

[39] Kusaba T., Lalli M., Kramann R., Kobayashi A., Humphreys B.D. (2014). Differentiated kidney epithelial cells repair injured proximal tubule. Proc. Natl. Acad. Sci. USA.

[40] Sun Y.B., Qu X., Caruana G., Li J. (2016). The origin of renal fibroblasts/myofibroblasts and the signals that trigger fibrosis. Differentiation.

[41] Li J., Deane J.A., Campanale N.V., Bertram J.F., Ricardo S.D. (2007). The contribution of bone marrow-derived cells to the development of renal interstitial fibrosis. Stem Cells.

[42] Simon-Tillaux N., Hertig A. (2017). Snail and kidney fibrosis. Nephrol. Dial. Transplant..

[43] Bai X., Geng J., Zhou Z., Tian J., Li X. (2016). Microrna-130b improves renal tubulointerstitial fibrosis via repression of snail-induced epithelial-mesenchymal transition in diabetic nephropathy. Sci. Rep..

[44] Qiao B., Johnson N.W., Gao J. (2010). Epithelial-mesenchymal transition in oral squamous cell carcinoma triggered by transforming growth factor-beta1 is snail family-dependent and correlates with matrix metalloproteinase-2 and -9 expressions. Int. J. Oncol..

[45] Wang S., Chen Q., Simon T.C., Strebeck F., Chaudhary L., Morrissey J., Liapis H., Klahr S., Hruska K.A. (2003). Bone morphogenic protein-7 (BMP-7), a novel therapy for diabetic nephropathy. Kidney Int..

[46] Lan A., Qi Y., Du J. (2014). Akt2 mediates TGF-β1-induced epithelial to mesenchymal transition by deactivating GSK3β/snail signaling pathway in renal tubular epithelial cells. Cell. Physiol. Biochem..

[47] Yokoyama K., Kamata N., Fujimoto R., Tsutsumi S., Tomonari M., Taki M., Hosokawa H., Nagayama M. (2003). Increased invasion and matrix metalloproteinase-2 expression by snail-induced mesenchymal transition in squamous cell carcinomas. Int. J. Oncol..

[48] Allaire A.D., Ballenger K.A., Wells S.R., McMahon M.J., Lessey B.A. (2000). Placental apoptosis in preeclampsia. Obstet. Gynecol..

[49] Cheng S., Pollock A.S., Mahimkar R., Olson J.L., Lovett D.H. (2006). Matrix metalloproteinase 2 and basement membrane integrity: A unifying mechanism for progressive renal injury. FASEB J..

[50] Tveitaras M.K., Skogstrand T., Leh S., Helle F., Iversen B.M., Chatziantoniou C., Reed R.K., Hultstrom M. (2015). Matrix metalloproteinase-2 knockout and heterozygote mice are protected from hydronephrosis and kidney fibrosis after unilateral ureteral obstruction. PLoS ONE.

[51] Grande M.T., Sanchez-Laorden B., Lopez-Blau C., De Frutos C.A., Boutet A., Arevalo M., Rowe R.G., Weiss S.J., Lopez-Novoa J.M., Nieto M.A. (2015). Snail1-induced partial epithelial-to-mesenchymal transition drives renal fibrosis in mice and can be targeted to reverse established disease. Nat. Med..

[52] Lovisa S., LeBleu V.S., Tampe B., Sugimoto H., Vadnagara K., Carstens J.L., Wu C.C., Hagos Y., Burckhardt B.C., Pentcheva-Hoang T. (2015). Epithelial-to-mesenchymal transition induces cell cycle arrest and parenchymal damage in renal fibrosis. Nat. Med..

[53] Galichon P., Hertig A. (2011). Epithelial to mesenchymal transition as a biomarker in renal fibrosis: Are we ready for the bedside?. Fibrogenes. Tissue Repair.

[54] Menon M.C., Ross M.J. (2016). Epithelial-to-mesenchymal transition of tubular epithelial cells in renal fibrosis: A new twist on an old tale. Kidney Int..

[55] Chen G., Chen H., Wang C., Peng Y., Sun L., Liu H., Liu F. (2012). Rapamycin ameliorates kidney fibrosis by inhibiting the activation of mTOR signaling in interstitial macrophages and myofibroblasts. PLoS ONE.

[56] Lindquist J.A., Mertens P.R. (2013). Myofibroblasts, regeneration or renal fibrosis—Is there a decisive hint?. Nephrol. Dial. Transplant..

[57] Falke L.L., Gholizadeh S., Goldschmeding R., Kok R.J., Nguyen T.Q. (2015). Diverse origins of the myofibroblast-implications for kidney fibrosis. Nat. Rev. Nephrol..

[58] Wolf G. (2004). New insights into the pathophysiology of diabetic nephropathy: From haemodynamics to molecular pathology. Eur. J. Clin. Investig..

[59] Yamamoto T., Nakamura T., Noble N.A., Ruoslahti E., Border W.A. (1993). Expression of transforming growth factor beta is elevated in human and experimental diabetic nephropathy. Proc. Natl. Acad. Sci. USA.

[60] Hills C.E., Bland R., Bennett J., Ronco P.M., Squires P.E. (2009). TGF-beta1 mediates glucose-evoked up-regulation of connexin-43 cell-to-cell communication in HCD-cells. Cell. Physiol. Biochem..

[61] Bottinger E.P., Bitzer M. (2002). TGF-beta signaling in renal disease. J. Am. Soc. Nephrol..

[62] Loeffler I., Wolf G. (2014). Transforming growth factor-β and the progression of renal disease. Nephrol. Dial. Transplant..

[63] Zhang Y., Zhang Q. (2009). Bone morphogenetic protein-7 and Gremlin: New emerging therapeutic targets for diabetic nephropathy. Biochem. Biophys. Res. Commun..

[64] Wang S.N., Lapage J., Hirschberg R. (2001). Loss of tubular bone morphogenetic protein-7 in diabetic nephropathy. J. Am. Soc. Nephrol..

[65] De Petris L., Hruska K.A., Chiechio S., Liapis H. (2007). Bone morphogenetic protein-7 delays podocyte injury due to high glucose. Nephrol. Dial. Transplant..

[66] Zeisberg M., Hanai J., Sugimoto H., Mammoto T., Charytan D., Strutz F., Kalluri R. (2003). BMP-7 counteracts TGF-beta1-induced epithelial-to-mesenchymal transition and reverses chronic renal injury. Nat. Med..

[67] Barnes J.L., Gorin Y. (2011). Myofibroblast differentiation during fibrosis: Role of NAD(P)H oxidases. Kidney Int..

[68] Hills C.E., Squires P.E. (2010). TGF-beta1-induced epithelial-to-mesenchymal transition and therapeutic intervention in diabetic nephropathy. Am. J. Nephrol..

[69] Inoue T., Umezawa A., Takenaka T., Suzuki H., Okada H. (2015). The contribution of epithelial-mesenchymal transition to renal fibrosis differs among kidney disease models. Kidney Int..

[70] Chen C.L., Chou K.J., Lee P.T., Chen Y.S., Chang T.Y., Hsu C.Y., Huang W.C., Chung H.M., Fang H.C. (2010). Erythropoietin suppresses epithelial to mesenchymal transition and intercepts Smad signal transduction through a MEK-dependent mechanism in pig kidney (LLC-PK1) cell lines. Exp. Cell Res..

[71] Zhang M., Fraser D., Phillips A. (2006). Erk, p38, and Smad signaling pathways differentially regulate transforming growth factor-beta1 autoinduction in proximal tubular epithelial cells. Am. J. Pathol..

[72] Nangaku M. (2013). Tissue protection by erythropoietin: New findings in a moving field. Kidney Int..

[73] Coldewey S.M., Khan A.I., Kapoor A., Collino M., Rogazzo M., Brines M., Cerami A., Hall P., Sheaff M., Kieswich J.E. (2013). Erythropoietin attenuates acute kidney dysfunction in murine experimental sepsis by activation of the β-common receptor. Kidney Int..

